# Tap the sap – investigation of latex-bearing plants in the search of potential anticancer biopharmaceuticals

**DOI:** 10.3389/fpls.2022.979678

**Published:** 2022-11-01

**Authors:** Oliwia Mazur, Sophia Bałdysz, Alicja Warowicka, Robert Nawrot

**Affiliations:** ^1^ Department of Molecular Virology, Institute of Experimental Biology, Adam Mickiewicz University, Poznań, Poland; ^2^ NanoBioMedical Centre, Adam Mickiewicz University, Poznań, Poland

**Keywords:** latex-bearing plants, anticancer activity, nanoparticles, natural products, cytotoxicity

## Abstract

Latex-bearing plants have been in the research spotlight for the past couple of decades. Since ancient times their extracts have been used in folk medicine to treat various illnesses. Currently they serve as promising candidates for cancer treatment. Up to date there have been several *in vitro* and *in vivo* studies related to the topic of cytotoxicity and anticancer activity of extracts from latex-bearing plants towards various cell types. The number of clinical studies still remains scarce, however, over the years the number is systematically increasing. To the best of our knowledge, the scientific community is still lacking in a recent review summarizing the research on the topic of cytotoxicity and anticancer activity of latex-bearing plant extracts. Therefore, the aim of this paper is to review the current knowledge on *in vitro* and *in vivo* studies, which focus on the cytotoxicity and anticancer activities of latex-bearing plants. The vast majority of the studies are *in vitro*, however, the interest in this topic has resulted in the substantial growth of the number of *in vivo* studies, leading to a promising number of plant species whose latex can potentially be tested in clinical trials. The paper is divided into sections, each of them focuses on specific latex-bearing plant family representatives and their potential anticancer activity, which in some instances is comparable to that induced by commonly used therapeutics currently available on the market. The cytotoxic effect of the plant’s crude latex, its fractions or isolated compounds, is analyzed, along with a study of cell apoptosis, chromatin condensation, DNA damage, changes in gene regulation and morphology changes, which can be observed in cell post plant extract addition. The *in vivo* studies go beyond the molecular level by showing significant reduction of the tumor growth and volume in animal models. Additionally, we present data regarding plant-mediated biosynthesis of nanoparticles, which is regarded as a new branch in plant latex research. It is solely based on the green-synthesis approach, which presents an interesting alternative to chemical-based nanoparticle synthesis. We have analyzed the cytotoxic effect of these particles on cells. Data regarding the cytotoxicity of such particles raises their potential to be involved in the design of novel cancer therapies, which further underlines the significance of latex-bearing plants in biotechnology. Throughout the course of this review, we concluded that plant latex is a rich source of many compounds, which can be further investigated and applied in the design of anticancer pharmaceuticals. The molecules, to which this cytotoxic effect can be attributed, include alkaloids, flavonoids, tannins, terpenoids, proteases, nucleases and many novel compounds, which still remain to be characterized. They have been studied extensively in both *in vitro* and *in vivo* studies, which provide an excellent starting point for their rapid transfer to clinical studies in the near future. The comprehensive study of molecules from latex-bearing plants can result in finding a promising alternative to several pharmaceuticals on the market and help unravel the molecular mode of action of latex-based preparations.

## Graphical Abstract

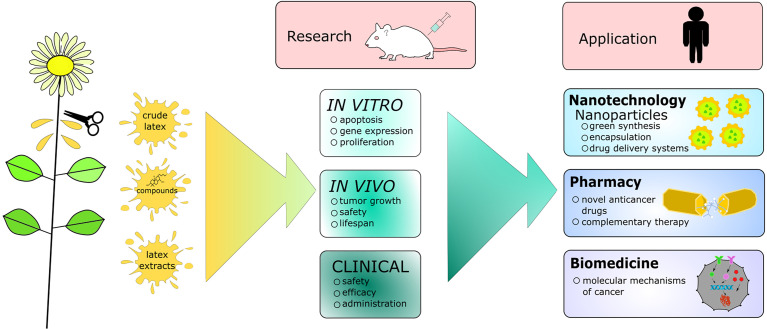

## Introduction

There are over 35,000 lactiferous species, from which the latex and its isolated compounds have been used in folk medicine for centuries (Konno et al., 2004; Almeida et al., 2014). One of the oldest pieces of evidence of use of medical plants as drugs is approximately 5000 years old and comes from a Sumerian clay slab from Nagpur. It includes recipes for approximately 250 latex-derived drugs from plants, including opium poppy. Nowadays preparations based on latex-bearing plants are still used and are commonly used especially in South America, where recipes based on the Farmacopéia Homeopática Brasileira (FHB) are applied to treat various types of cancers (Aquino et al., 2021). Despite their wide use and approval of ethical committees, there are still questions regarding the effectiveness and potential toxicity of such preparations. In several cases their possible cytotoxic effects have not been extensively studied. This provides an opportunity for the scientific community to decrease the knowledge gap on latex-bearing plants and their effects on human health.

Plant latex is often referred to as milky sap, or just sap. It exudes from the plant immediately after mechanical damage or injury and often coagulates after air exposure (Cho et al., 2014; Nawrot, 2017). For many plant species it is very viscous and of different colors - from white to yellow/orange or non-transparent (Konno, 2011; Nawrot et al., 2016). Plant latex is an exudate of specialized cells called laticifers, which comprises a separate tissue associated with phloem. Laticifers are elongated and present in different organs throughout the whole plant. During plant growth laticifers develop and their cytoplasm often degrades and mixes with their vacuole content. This cytoplasmic-vacuole mixture of laticifers forms the latex (Konno, 2011; Zhou and Liu, 2011), which is a complex emulsion composed of diverse low-molecular compounds and proteins. These compounds vary amongst plant species and their functions are mainly unknown. Small molecular chemicals are summarized as natural products or secondary metabolites, such as various alkaloids, terpenoids, starches, sugars, oils, tannins, gums and others (Konno, 2011; Cho et al., 2014).

Laticifers develop in many unrelated plant orders with diverse morphology. They can be divided into two sub-groups: articulated laticifers and non-articulated laticifers (Agrawal and Konno, 2009; Agrawal and Hastings, 2019). Articulated laticifers develop from multiple cells, are elongated fig and may be either anastomosing or non-anastomosing. Anastomoses can comprise a large network of continuous cytoplasm throughout the whole plant (Hagel et al., 2008; Nawrot, 2020). This type is present e.g. in *Papaver somniferum*, and *Hevea brasiliensis*. Non-articulated laticifers develop from single cells, are multinucleate and may be branched or unbranched. The latter are present in *Catharanthus roseus* or *Cannabis sativa* (Hagel et al., 2008; Pickard, 2008).

**Figure 1:**
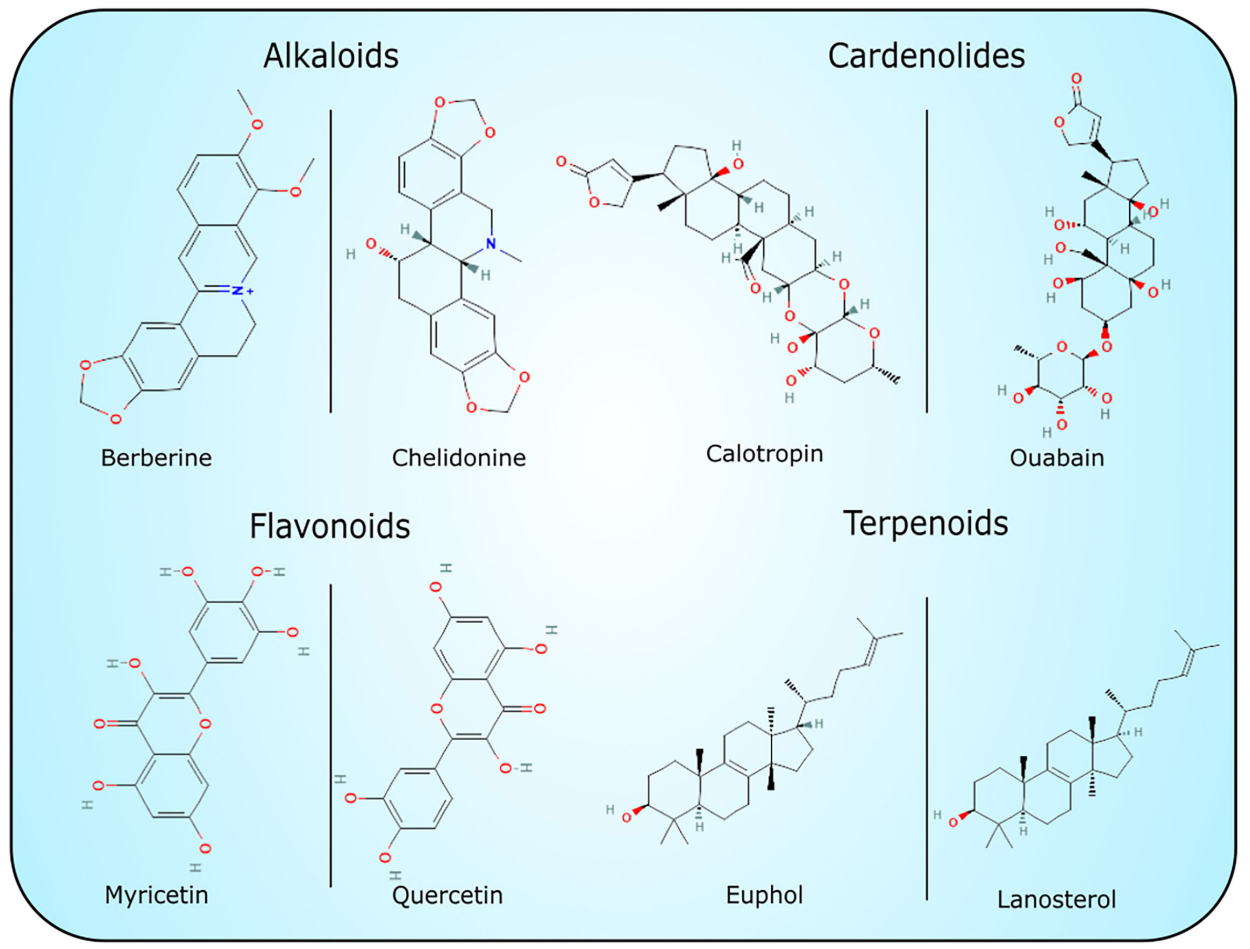
Chemical structures of selected low weight molecular compounds with potential cytotoxic activity.

The exact function of laticifers and latex is unknown. However, several hypotheses have been set forth to explain their possible functions. The latex has evolved in numerous plant families and orders, which suggests that it represents a highly convergent trait, which presents common advantages in latex-borne defenses (Hagel et al., 2008; Konno, 2011). Plant latex contains highly concentrated defense substances and is exuded immediately after damage, which in turn enables the defense molecules to act exactly at the point of damage against herbivores and different pathogens (Nawrot, 2017; Nawrot, 2020). The range of bioactive specialized metabolites present in the latex, including alkaloids, terpenoids, lignans, cannabinoids, cardiac glycosides, and tannins show numerous biological activities, including anticancer, antiproliferative, anti-inflammatory, antioxidant, antimicrobial, antiparasitic and insecticidal (**Figure 1**) (Konno, 2011; Upadhyay, 2011; Mithöfer and Boland, 2012; Lee et al., 2013). Despite the presence of low-molecular compounds, the activity of macromolecular complexes (proteins) is also very important. Most proteins identified in plant latex belong to pathogenesis-related (PR) proteins. Their expression is induced by different viral, bacterial and fungal pathogens (Sels et al., 2008). They have various structures and are classified into 17 families according to their various activities - antifungal (e.g. chitinases), antiviral (nucleases), antibacterial (defensins, thionins, lipid transfer proteins) and others (Warowicka and Nawrot; van Loon et al., 2006; Sels et al., 2008; Fister et al., 2016). Recently, a model of antiviral response in *Chelidonium majus* was proposed which included the action of two latex proteins: major latex protein (MLP) and glycine-rich protein (GRP), constituting the third line of immediate defense response with exuding latex (Nawrot, 2017).

Many species of other latex-bearing plants are important in the global economy and pharmacy. Plant latex comprises a “treasury” of defense substances, which could be used as medicines, toxins, pharmaceuticals, immune allergens, pesticides and other bioproducts important for human use (Hagel et al., 2008; Upadhyay, 2011; Gracz-Bernaciak et al., 2021). To assure the safety and effectiveness of treatments from plant latex, numerous *in vitro* and *in vivo* studies have been conducted. The examination of the activity of crude latex, its polar or non-polar fractions and most importantly, individual compounds, can uplift the discovery of new pharmaceuticals. Additionally, the careful studies of recipes used in folk medicine can help to unravel their mode of action as well as confirm their effectiveness. Although plant latex can serve as an inexhaustible source of therapeutics for several diseases, cancer remains in the pharmaceutical studies spotlight. Researchers estimate that in 2022 there will be 1.9 million new cancer diagnoses, with approximately 609,360 cancer-related deaths in the United States alone (Siegel et al., 2022). The scale of the problem makes the screening for novel anticancer pharmaceuticals a priority. Apart from *in vitro* and *in vivo* studies, several clinical trials have been carried out to test natural products such as curcumin, turmeric, epigallocatechin gallate (EGCG), genistein and quercetin in treatment of cancer. Some of those trials have been successfully completed and compounds such as resveratrol and artemisinin or even whole plant extracts (green tea, American ginseng root (LEAG)) are proving to be effective in cancer treatments (Dehelean et al., 2021). It is worth noting that several routinely used chemotherapeutics, including paclitaxol, vinca alkaloids (vinblastine, vincristine), podophyllotoxins and irinotecan, are all of plant origin (Wongprayoon and Charoensuksai, 2018).

Naturally derived compounds have been shown to have multi-level effects on cancer. Most importantly, they induce apoptosis, which is a central regulator of cell homeostasis crucial in tumorigenesis prevention. Based on the available up-to-date data it might be assumed that targeting the apoptosis event can result in effective treatment of cancer therapy (Elmore, 2007). Additionally, plant-based compounds can also affect the expression of cell cycle regulating genes (Zheng et al., 2019; Jang et al., 2021). Downstream *in vivo* studies showed positive outcomes in animal models, including tumor growth inhibition, lowered tumor volume as well as increased lifespan.

In this review we provide mostly *in vitro* and *in vivo* data regarding the anticancer activity of latex-bearing plants, their extracts or individual compounds. We discuss the multi-level activity of latex from several widely distributed latex-bearing plant families, including Apocynaceae, Euphorbiaceae, Moraceae and Papaveraceae. The mentioned data mainly focuses on the molecular mode of action of the extracts and their effectiveness as well as safety data. Additionally, we briefly describe the methodology behind the cytotoxicity studies and provide a description of the nanoparticles-based research, which ensures more targeted delivery of the plant compounds and novel biotechnological development in that field. Finally we summarize the article by putting forth future perspectives in the research on the topic of anticancer properties of latex-bearing plants. The most significant aspects of the presented review are illustrated in the graphical abstract (Graphical Abstract).

## Anticancer properties of latex-bearing plant extracts

### Family: Apocynaceae

Latex-bearing representatives of the Apocynaceae family have provided an ample source of compounds and extracts for medicinal purposes. They have exceptionally rich latex, known for its various cardenolides. Plants from this family grow all around the world, including the African continent, the Arabian Peninsula, Western Asia, South America and Australia.

*Calotropis procera* is a perennial Asian shrub, which grows on depleted soils and adverse climate conditions (Kaur et al., 2021). Due to its white, milky latex, rich in biologically active compounds, including a high cardenolide content and proteins (**Table 1**), it has been used in folk medicine in treatment of several diseases (Mascolo et al., 1988; Qasim Khan and Malik, 1989; Kumar and Arya, 2006). *C. procera* latex is chemically distinct from Euphorbiaceae and Papaveraceae plants, since it lacks phenols, saponins and alkaloids, which are present only in its non-latex organs and tissues (Pant and Chaturvedi, 1989).

**Table 1:** Biological compounds with potential anticancer activity present in the latex of Apocynaceae family representatives.

Plant species	Compounds present in the latex		Reference
	Low molecular weight molecules	Proteins	
*Asclepias curassavica*	Alkaloids (quinines) Carboxylic acids Cardenolides (calotropin, calactin, asclepion, uscharidin, uzarin, calotroposide, uscharidin) Coumarins Flavonols (flavonoid glycosides) Phenols (tannin, lignans) Saponins Steroids Terpenoids		(Al-Snafi, 2019)
*Asclepias fruticosa*	Glycosides (gomphoside, afroside, calactin, voruscharin)		(Cheung et al., 1983)
*Asclepias vestita*	19-oxo cardenolide glycosides with characteristic 5(6) double-bonds		(Cheung et al., 1989)
*Aclepias syriaca*	Cardiac glycosides Flavonoid glycosides Glycosylated megastigmane Lignans Pentacyclic triterpenes Phenols (phenyl-ethanoid)		(Araya et al., 2012)
*Calotropis procera*	Cardenolides (alotoxin, uscharin, voruscharin, uscharidin, 2’-oxovuruscharin, calotropin, calotoxin, calactin) Flavonoids (quercetin) Hydrocarbons Steroids Tannins Triterpenoids	Chitinases UNBS1450 Cyclopeptides Latex proteins (CpPII)	(Mascolo et al., 1988; Pant and Chaturvedi, 1989; Qasim Khan and Malik, 1989; Van Quaquebeke et al., 2005; Juncker et al., 2009; Mohamed et al., 2011; Cornelius et al., 2013; Jucá et al., 2013; Viana et al., 2017; Oliveira et al., 2021)
*Himatanthus sucuuba*	Terpenoids (plumericin, isoplumericin and lactones/apigenin)		(Au et al., 2006; Silva et al., 2010)

The activity of latex can vary greatly between different extracts used depending on their polarity. In the studies of the activity of *C. procera* various latex extracts, cytotoxic activity was noted for hydrophobic fractions, including chloroform extract, which appeared to be the most toxic against human colon cancer (HCT-116), human glioblastoma (SF-295), human ovarian carcinoma (Ovcar-8) and acute promyelocytic leukemia (HL-60) cell lines (Jucá et al., 2013; Said et al., 2014).However, other studies had presented the high cytotoxic activity of multiple hydrophilic fractions including: ethyl acetate, ethanolic and methanol extracts. The dried latex ethanolic extracts showed apoptosis induction and cytotoxicity towards the breast cancer cell line MCF-7 and epithelioid cervix carcinoma HeLa cells (Chowdary et al., 2011). In the shrimp larvae assay, ethyl acetate extracts were proven highly cytotoxic, with higher LC50 levels when extract were obtained from the leaves and stem (Oladimeji et al., 2009; Said et al., 2014). The activity of ethyl acetate and acetate fraction of extracts from *C. procera* were also shown to be toxic towards HCT-8 colon and B-16 melanoma cancer cell lines (Magalhães et al., 2010). Sea urchin egg development assay showed an inhibitory effect of several *C. procera* latex fractions towards egg cell division in a dose dependent manner, probably due to inhibition during cleavage related to DNA and protein synthesis, as well as microtubule assembly and RNA synthesis (Magalhães et al., 2010). Another hydrophilic fraction, the methanolic extract of dried latex (DL) from *C. procera*, had a pronounced effect on MCF-7 and epithelioid cervix carcinoma HeLa cell line (Chowdary et al., 2011), as well as on Huh-7 hepatoma carcinoma cell lines, non-transformed mouse liver cells AML12 and fibroblast-like COS-1 cell lines, resulting in approximately 90% cell death of cancerous cells. This suggests the selectivity of DL extracts, which is possibly caused by the altered metabolic status of cancerous cells. Gene expression analysis had shown no changes in Bcl-2 and caspase 3 expression patterns, both of which are markers of mitochondria-related apoptotic death. Due to the noticeable increase in cellular DNA fragmentation in the cells, a different source of apoptotic events, apart from mitochondrial pathways, has been suggested (Choedon et al., 2006). The follow up studies on X15-myc transgenic mice model of hepatocellular carcinoma have shown the *in vivo* chemopreventive effect of *C. procera* dried latex (DL). Examination of mice livers after 20 weeks of oral administration of DL (400 mg/kg) showed hypochromia, necrosis, nuclear atypia and loss of sinusoidal architecture. Although the slight disruption in cellular integrity and architecture was seen, the treatment with DL was able to protect mice from malignant changes. Additional measurements of the vascular endothelial growth factor (VEGF), an angiogenesis marker, was shown to be significantly decreased in X15-*myc* mice (Choedon et al., 2006).

Apart from whole latex fractions or its extract, singular secondary metabolites have been extensively studied. Molecules such as quercetin, isorhamnetin and azaleatin were found to be cytotoxic. Quercetin was found to be the most toxic, probably due to the presence of free phenolic OH groups and no methoxy substitutions (Mohamed et al., 2011). Cardenolies, especially abundant in Apocynaceae latex, presented various cytotoxic activity towards non-small lung cancer A549 and HeLa cell lines. Throughout the individually tested compounds, aflatoxin and calactin presented higher cytotoxic effects against both cell lines than their non-glycosidic derivatives. It has been proposed that the presence of the double linked six-membered ring sugar moiety in those compounds can presumably enhance the anticancer properties of cardiac glycosides (Cornelius et al., 2013). 2’-Oxovuruscharin, a cardenolide isolated from *C. procera* latex, had gained attention due to its ability to reduce overall growth of different human cancer cell lines in levels comparable to that of Taxol and greater than SN-38, both of which are commonly used chemotherapy drugs (Van Quaquebeke et al., 2005). The aforementioned compound has given rise to a hemi-synthetic cardenolide, UNBS1450, which has been shown to have an antiproliferative effect and induce cell death through its inhibition of sodium pumps.

This inhibition is presumed to occur due to its double-linked sugar moiety and its steroid core structure. The UNBS1450 had also shown an inhibitory effect on A549, as well as human astrocytoma (U373-MG) cells, through interference in the NF-κB signaling pathway and not enabling daughter cells division, respectively. Other studies connected cell death induced by UNBS1450 to autophagy due to the presence of PARP cleavage, vacuole formation and downregulation of the Hsp70 heat shock protein. Therefore it was assumed that cell death of NSCLC after administration of UNBS1450 was not caused by apoptosis (Mijatovic et al., 2006; Juncker et al., 2009). An autophagy process was also noted when testing UNBS1450 on human prostate cancer cell lines (Mijatovic et al., 2008) as well as on glioblastoma lines, with a notable increase of beclin-1 and LC3 autophagy-related gene markers (Lefranc et al., 2008). *In vivo* studies showed increased survival of A549 orthotopic xenograft-bearing nude mice after administration of UNBS1450. In comparison to other cardenolides, including ouabain, digoxin and 2-oxovoruscharin, a 10 mg/kg dose of UNBS1450 showed increased maximum tolerated dose (MTD) values, which lengthened mice survival (Juncker et al., 2009). Additionally, other than secondary metabolites, proteins present in *C. procera* latex were studied in regards to their cytotoxicity. The activity of these proteins and their anticancer effects both *in vitro* and *in vivo* have been summarized in **Table 2**.

**Table 2:** Potential anticancer activity of *Calotropis procera* latex proteins in *in vitro* and *in vivo* models.

Protein name	Anticancer effect	Model	Reference
Chitinases (LPp1-P1-P6)	Increase in expression of pro-inflammatory cytokines	*In vitro* (SF295, OVCAR-8, HVCT-116)	(Viana et al., 2017)
Chitinase (LPp1-P4)	Decrease of TNF-α and IL1-β Increased amount of pro-inflammatory cytokine IL-6	*In vivo* (healthy mice)	(Viana et al., 2017)
CpPII latex protein	Improvement functioning of hepatic mitochondria Increase of cell viability, basal oxygen consumption rate (OCR) and ATP-linked respiration Increase of mitochondrial membrane potential (MMP) and proton leak Increase in expression of enzymes involved in metabolic oxidative pathways, as well as proteins in mitochondrial complexes Reduced lactate concentration in HepG2 cells	*In vitro* (HepG2)	(Oliveira et al., 2021)
Laticifer proteins (LP) fraction	Apoptosis-inducing effect Inhibition of DNA synthesis through intercalation or interaction with DNA	*In vitro* (SF295, MDA-MB-435, HL-60)	(Soares de Oliveira et al., 2007)

Singular studies have been conducted with the use of other plants from the Apocynaceae family. *Aloe niebuhriana* showed weak cytotoxic activity towards MCF-7, human liver cancer HepG2 and HCT-116 cell lines. However, studies of other *Aloe* species have shown a similar cytotoxic effect towards normal human keratinocytes (Moharram et al., 2020). Hence, this cytotoxic effect may not be restricted to cancer cells. The latex from *Pergularia tomentosa* was studied for its effect on several cancer cell lines, including HCT-116, HepG2 and MCF-7, *via* the crystal violet staining method (Ads et al., 2021). The methanol extract from the latex showed a strong cytotoxic activity towards HCT-116 and HepG2, whereas the effect towards MCF-7 was much weaker. The crude latex extract showed an even weaker effect towards all cell lines. The cytotoxic effect is presumed to be attributed to flavonoids and phenolic compounds present in the latex. *H. speciosa* latex showed no cytotoxic effects towards *A. cepa* cells (Ribeiro et al., 2016), which confirmed the results obtained in an *in vitro* study on mice fibroblasts using a MTT, red neutral test and comet assay *(* Almeida et al., 2014 *)*. The cytotoxic activity has also been confirmed for *H. sucuuba* (Silva et al., 2010). The analysis of the latex revealed the presence of plumericin, isoplumericin and lactones, which can cause DNA damage. Latex screening was carried out using a Rad52 repair-deficient mutant strain of *Saccharomyces cerevisiae* due to its association with the DNA repair pathway and the ability of compounds to interact with topoisomerase I. Iridoid lactones present in the hexane fraction of the latex were found to be more effective in repair-deficient strains, which suggests the DNA damaging activity of the compounds and the inhibitory effect on the topoisomerase II (Silva et al., 2010). Contrastingly, a non-cytotoxic effect of the latex of *H. succuba* was observed in 2 colon tumor cell lines. (Herrera-Calderón et al., 2021). Isolated compounds from *H. succuba* latex, including apigenin, showed antitumor properties (Herrera-Calderón et al., 2021). A study carried out in Brazil involved the *H. bracteatus* (A.DC.) Woodson plant, whose latex had been used prior to treat external ulcers and tumors (da Silva et al., 2016; Herrera-Calderón et al., 2021). The chloroform partition from the ethanolic extract from its latex showed cytotoxic activity against several tumor cell lines: mouse melanoma, human hepatocellular carcinoma, human promyelocytic leukemia, human chronic myelocytic leukemia. Moreover, the extract showed a high growth inhibition rate against human hepatocellular carcinoma and human promyelocytic leukemia cells (da Silva et al., 2016).

The Apocynaceae consist of several subfamilies, one of which, *Asclepiadaceae*, representatives are spread worldwide, from the Strait of Magellan in South America to the northern hemisphere in Canada and Siberia. The latex from plants from this family contains several secondary metabolites (Araya et al., 2012), however, the latex content varies greatly between species.

*Asclepias syriaca* L. is mainly used as an industrial crop in the United States (Abbott et al., 2002). Its latex has been also used in traditional medicine to treat asthma, venereal diseases, kidney stones, wart removal and edema (Hamel and Chiltoskey, 1975; Moerman, 2009). Five compounds from the methanolic extract from dried plant material of *A. syriaca* had a cytotoxic effect on breast cancer cell lines (MCF-7, T47D, Sk-Br-3), with IC50 values being comparable to those of other cardenolides including digoxin, digitoxigenin and ouabain. Interestingly, the tested compounds showed selectivity in action towards cancer cells (Araya et al., 2012).

Ethyl acetate extracts of *Asclepias curassavica* (EAAC) showed an antitumor effect on A549 and non-small cell lung cancer NIC-H1975 cell lines through a real-time cell detection system. EAAC was cytotoxic towards both cell lines, in a time and dose dependent manner. Additional flow cytometry showed the promotion of cells into an early stage of apoptosis as well as signs of nuclear fragmentation, chromatin condensation and activation of caspase 3/8/9 and a consequent PARP cleavage in affected cells, showing the involvement of death receptor pathways, as well as mitochondrial pathways, in the apoptosis process. Downregulation of antiapoptotic proteins was also noticed in EAAC treated samples (**Table 3**). Western blot analysis showed activation of p38 and JNK MAPK pathways, which are involved in the induction of apoptosis (Zheng et al., 2019). Additionally to an *in vitro* investigation suggesting the strong multilevel anticancer activity of EAAC, an *in vivo* on NIC-H1975 BALB/c-nu/nu mice showed lowered tumor weight and volume in EAAC treated mice, as well as significant tumor growth inhibition (Zheng et al., 2019).

**Table 3:** Changes in expression patterns of cancer-related genes post administration of plant latex extracts or its compounds *in vitro* or *in vivo*.

Plant species	Effect in cancer-related gene expression along with corresponding proteins and pathways	Tested biological extract or compound	Model	Reference
*Asclepias curassavica*	Activation of caspase 3/8/9, p38 and JNK MAPK pathways involved in the induction of apoptosis Downregulation of antiapoptotic proteins XIAP, survivin, Bcl-2 and Mcl-1	Ethyl acetate extract	*In vitro* (NIC-H1975); *in vivo* (NIC-H1975 tumors in BALB/c-nu/nu mouse)	(Zheng et al., 2019).
*Chelidonium majus*	Decreased levels of mitotic-slippage-associated proteins Increase in phosphorylation of histone H3 involved in the structure of chromatin in eukaryotic cells Increase of p53 and p21 protein levels, which induce cleavage of caspase-3 through the GADD45a and p53 pathways Upregulation of p53 tumor suppressor	Chelidonine	*In vitro* (MIA PaCa-2)	(Qu et al., 2016; Jang et al., 2021)
*Calotropis procera*	Increased citrate synthase (CS), pyruvate dehydrogenase kinase 4 (PDK4) and AMPK pathway activity involved in metabolic oxidative pathways Increased levels of proteins in mitochondrial complexes I, III and V Reduced lactate and intracellular ROS levels	Latex proteins (CpPII)	*In vitro* (HepG2)	(Oliveira et al., 2021)
	Increased expression of beclin-1 and LC3 autophagy markers	UNBS1450, a hemi-synthetic derivative of 2’-Oxovuruscharin	*In vitro* glioblastoma lines (GBM clinical samples, normal brain tissue, human GBM cell line)	(Lefranc et al., 2008)
	Decrease of cdc34 levels- an enzyme connected to IκB α ubiquitination Downregulation of Hsp70, which is responsible for lysosomal membrane permeabilization and cell death through autophagy	UNBS1450	*In vitro* (A549 NSCLC)	(Mijatovic et al., 2006; Juncker et al., 2009)
	Increase of cytokines (IL-6, IL-1-β,TNF-α) and iNOs enzyme levels	Chitinases (LPp1-P1-P6)	*In vitro* (SF295, OVCAR-8, HVCT-116)	(Viana et al., 2017)
*Euphorbia helioscopia* L.	Downregulation of MMP-9 expression involved in cancer cell migration, invasion and degradation of the extracellular matrix	Ethyl acetate extract	*In vitro* (SW480, SGC-7901, SMMC-7721, HepG2 and BEL-740)	(Wang et al., 2012)
	Increase of the nm23-H1 protein Reduction of Bcl-2, CyclinD1 and MMP-9 proteins classified as anti-apoptotic factors Upregulation of bax pro-apoptotic factor and caspase-3 involved in several apoptosis signaling transduction pathways		*In vivo* (nude mice hepatocellular carcinoma xenografts)	(Cheng et al., 2015)
*Euphorbia tirucalli*	Downregulation of antiapoptotic proteins including XIAP, BCL-X, BAX, Trail R1/DR4, BAX and Trail R1/DR5 Increased levels of Thioredoxin-1, P21 CIP1 and SOD2 Reduced expression of Bcl-2 and NF-kB1 Slight upregulation of P52 (S15) and P53 (S46)	Euphol	*In vitro*, glioma cell lines (GAMG, SF188, RES259, SW1783, UW479, RES186)	(Silva et al., 2019)
	Decrease of GPx4 and MnSOD expression Increase in expression of antioxidant-related genes (CAT)	Aqueous extract	*In vitro* (human peripheral blood leukocytes)	(Waczuk et al., 2015)
*Ficus carica*	Downregulation of CREB, GSK-2α/β,	Leaf latex	*In vitro* (MDA-MB-231)	(AlGhalban et al., 2021).
	Downregulation of HPV oncoproteins (E6 and E7) Increased expression of p53 and Rb tumor suppressors Misplacement of ki67 proliferation marker protein	Crude latex	*In vitro* (HeLa, CaSki)	(Ghanbari et al., 2019)
	Upregulation of ADPRTL1 expression, engaged in the base excision repair pathway	Fig fruit latex	*In vitro* (SMMC-7721,U251,L02)	(Wang et al., 2008)
*Ficus salicifolia*	Upregulation of CREB, GSK-2 α/β, AMPka, ERK involved in cell proliferation and cell attachment	Leaf latex	*In vitro* (MDA-MB-231)	(AlGhalban et al., 2021).
*Ficus religiosa*	Downregulation of Bcl-2, Nrf2 Downregulation of p53 expression (HCT-116) Upregulation of caspase-3 gene and Rel A (P65) Upregulation of p53 expression (IMR 32)	Extract containing flavonols (quercetin and myricetin) and phyrosterols (stigmasterol and β-sitosterol)	*In vitro* (IMR 32, HCT-116, MDA MB 231)	(Saida et al., 2021)

### Family: Euphorbiaceae

The study of cytotoxic effects of plant latices has also reached the Americas; tropical latex-bearing plants have been studied from Ecuador, Puerto Rico and Guadelupe (Guerrero and Guzmán, 2004). Representatives of the *Euphorbia* genus were used to treat external sores, warts, as well as cancer due to their anti-inflammatory, antiviral and analgesic activities (2015; Akihisa et al., 2001; Dutra et al., 2012; Silva et al., 2018). The Euphorbiaceae preparations based on Farmacopéia Homeopática Brasileira (FHB) guidelines are still commonly used. Although many species of this genus have been studied, it is worth mentioning that their latex can vary greatly in terms of content and therefore can possess different biomedical properties (**Table 1**) (Cataluna and Rates, 1997).

One of the best studied plants from this genus, a succulent *Euphorbia tirucalli*, grows in the tropical and subtropical climate and has well documented toxic activity towards mammals (dogs, cats, mice and rats) and fish (Neuwinger, 2004; Silva et al., 2007; Botha and Penrith, 2009; Kumar et al., 2010; Machado et al., 2016). Recently several articles have been published testing the potential anticancer activity of *E. tirucalli*, which is based on the abundance of sterols and terpenes in the latex (**Table 4**).

**Table 4:** Biological compounds with potential anticancer activity present in the latex of *Euphorbiaceae* family representatives.

Plant species	Compounds present in the latex		Reference
	Low molecular weight molecules	Proteins	
*Croton celtidifolius*	Phenolic compounds		(Biscaro et al., 2013)
*Euphorbiaceae tirucalli*	Flavonoids (kaempferol and quercetine) Polyphenols (gallic acid derivatives, chlorogenic acid, caffeic acid) Sterols Terpenes (taraxasterol, tirucallol, tigliane, ingenol, ingenane euphol and alpha-euphorbol)		(Newbold and Spring, 1944; Cataluna and Rates, 1997; Khaleghian et al., 2010; Waczuk et al., 2015)
*Euphorbia arabica;* *Euphorbia bupleurifolia;* *Euphorbia enopla;* *Euphorbiagorgonis*	Flavonoids Glycosides Phytosterols Triterpenoids		(Tebogo Michael Mampa et al., 2020)
*Euphorbia horrida indigeneous; Euphorbia horrida* var.	Flavonoids Glycosides Pentoses Phytosterols Polyphenols (tannins) Quinones (anthraquinones) Triterpenoids Saponins		(Tebogo Michael Mampa et al., 2020)
*Euphorbia neriifolia*	Phenols Amines Terpenoids (euphol)		(Upadhyaya et al., 2017)
*Euphorbia trigona*		Lectins	(Goldstein et al., 1980)
*Euphorbia umbellata*	Aliphatic compounds (oxygenated steroids) Terpenoids (euphol, lanosterol, tirucallol, cycloartenol, lupeol, taraxasterol, esters of phorbol)		(Cruz et al., 2020)
*Euphorbia heliscopia* L.	Flavonoids Flavonoids (quercetin) Terpenoids		(Wang et al., 2012)
*Jatropha curcas*	Coumarins Lignanes Phenolic compounds Phytosterols Saponins Tannins Terpenoids (curusone B, C, D and E, spirocurcasone, acetoxyjatropholonejatropholone, multidione, 4E-jatrogrossidentadion)	Curcin	(Naengchomnong et al., 1986; Gübitz et al., 1999; Lin et al., 2003; Muangman et al., 2005; Liu et al., 2009; Aiyelaagbe et al., 2011; Ahmed et al., 2020)

*E. tirucalli* latex have been acting cytotoxic against several cancer cell lines including lymphoma (Daudi), murine melanoma (B16F10) and HL-60 even when samples were collected throughout Brazil and differ geographically (Caxito et al., 2017). *E. tirucalli* has also been effective against HCT-116 cells, colorectal adenocarcinoma Caco-2-cells, B16F10 cells as well as canine cell lines, which are prone to develop many types of neoplasms. The highest latex dilutions (5-9) had an inhibitory effect in the case of CBMY canine melanoma cell lines as well as in human melanoma SK-MEL-28 cell lines. The U-shaped curve of cell viability, observed mainly in-human melanoma cells, suggests the hormesis effect in which a dose response of a cell or organism to a substance is biphasic. This effect means that cells benefit from low-dose stimulation by activation of the cellular stress response, outweighing the toxicity and suffer from a toxic or inhibitory effect when a high-dose of the agent is present (Mattson and Cheng, 2006; Khaleghian et al., 2010; Archanjo et al., 2016; Altamimi et al., 2019; Brunetti et al., 2019). Further *in vivo* investigations of *E. tirucalli* latex had variable outcomes. In the melanoma cancer model, induced using B16F10 cells inoculated into the tail vein of C57BL/6 mice the results were promising. Merely a 14-day treatment with a latex dilution was able to significantly reduce the volume of nodules in mice lungs resulting in only few melanoma colonies and no signs of congestion and hemorrhage within the lungs as well as no toxic alterations in kidneys, spleen or liver (Brunetti et al., 2019). Other studies had presented E. tirucalli aqueous latex solution to act pro-angiogenic through increase in neoangiogenesis and promotion of formation of vascular networks in chorioallantoic membranes (CAMs) of fertilized chicken eggs (Bessa et al., 2015).

As whole crude latex studies give us a general understanding of how latex expresses biomedical properties, when designing potential anticancer biopharmaceutical it is more preferable to focus on one particular molecule. One of the triterpenoids present in *E. tirucalli* latex, euphol, have been proven to act exceptionally well in several studies. It was proven to be highly effective, even in low doses, against GAMG and RES259 glioma lines, as well as moderately towards pediatric glioma cell lines (Silva et al., 2019). A human apoptosis and cell stress proteome array showed the downregulation of a majority of proteins in GAMG cell lines (**Table 3**) after euphol administration, as well as an increase in LC3-II expression and the development of fractional volume of acidic vesicular organelles (AVOs), which suggests the involvement of autophagy cell death related processes (Silva et al., 2019). An *in vivo* assay testing the activity of euphol from *E. tirucalli* had been used to evaluate the number of vessels formed around tumors from euphol-treated cells. A significant decrease in vessel numbers compared to the control suggested that euphol increased the angiogenesis processes (Silva et al., 2019). Although the majority of studies report positive outcomes of latex administration, with the reduced viability of cancer cells, it is also crucial to note the results of assessing the *E. tirucalli* genotoxicity. The evaluation of genotoxicity of *E. tirucalli* on human leukocytes has shown that addition of 10% extracts resulted in an increased proliferation of the cells. Latex extracts increased the frequency of micronuclei formation along with chromosome damage including chromosomal aberrations. (Machado et al., 2016). An additional study revealed a significant increase in oxidative stress response enzymes (**Table 3**) (Waczuk et al., 2015). In support of the previous study, administration of *E. tirucalli* extract resulted in significant toxicity (up to 30% cell viability reduction) as well as DNA damage. Based on the results of this study it can be assumed that *E. tirucalli* extracts were able to cause cytotoxic and genotoxic effects as well as changes in the expression of antioxidant related genes (Waczuk et al., 2015).

Another crucial factor when testing the biological substances, is testing them in the form that they have been used for several centuries. *E. tirucalli* latex was analyzed in human MV3 melanoma cells by using ultra diluted latex. The MTT assay absorbance results showed biological activity in all cell samples treated with ultra-diluted solutions, as well as in the control 5% tincture solution and cisplatin, which is a commonly used chemotherapeutic, however the cytotoxic effects for all dilutions did not exceed 60% (Silva et al., 2021). A similar study focused on the effects of diluted latex from *E. tirucalli* on the glycolytic metabolism and viability of MCF7 human breast cancer and MelanA line non-tumoral melanocyte cell lines. Due to the reduction of cell viability induced by the solvent (ethanol) alone it could not be concluded whether *E. tirucalli* was cytotoxic, however, its solutions were able to modify the glycolytic and mitochondrial metabolism in MCF7 cell lines (Aquino et al., 2021). Therefore the effectiveness of *E. tirucalli* latex preparations cannot be fully confirmed.

Another species of the Euphorbiaceae family that has been extensively studied is *Euphorbia umbellata*. *E. umbellata* is used in Brazilian folk medicine in a form of garrafada, which is a mixture containing crude latex diluted with water (Bariani Ortêncio, 1997). Its latex is exceptionally rich in diterpenes, which have been already proven to act against B16F10. The dichloromethane (DIF) extract of *E. umbellata*, which is rich in non-polar compounds, presented high cytotoxicity, lowering the viability of cancer cells without affecting non-cancerous ones (Wang et al., 2012; de Andrade et al., 2021). In other study, both non-polar (ethanol) as well as polar (hexane, dichloromethane) fractions was proven to be cytotoxic against HeLa, colorectal cancer HRT-18 and leukemia (HL-60, K-562 and Jurkat) cell lines. On the other hand, additional apoptotic and necrotic analysis showed no differences in the amount of necrotic cells. Flow cytometry showed cell cycle arrest, increase in DNA fragmentation, increase in cell populations in the Go/G1, whilst reducing the amount of cells in the S and G2/M phase. Only when exposed to high concentrations (80 μg/ml) of hexane fraction did the control cells have reduced viability. Activation of caspase 3 and 7 was also noted in cells towards which the highest concentration of latex was used. Hexane extract of *E. umbellata* has also been shown to promote apoptosis in leukemic cells (Luz et al., 2016; Cruz et al., 2020). However, in an *in vivo* study including a 15-day treatment of mice with *E. umbellata* extracts did not result in a significant reduction of tumor volume compared to the control group. Liver observations revealed the presence of lymph and liver nodes along with black spots in the spleen, suggesting metastasis (de Andrade et al., 2021).To further assess the cytotoxicity of this plant a test measuring the frequency of micronuclei in mice bone marrow cells was carried out (Melo-Reis et al., 2011). Measurements of the polychromatic and normochromatic erythrocytes ratio showed a significant difference between the control and studied group - the use of latex showed a reduction in the ratio in mice treated with a dose of latex equal to or higher than 30 mg/kg (Melo-Reis et al., 2011). These results led to believe that these doses have a cytotoxic effect on mice bone marrow. Given that this plant has been extensively used in treatment of several different tumors the potential harm towards the body of a patient must be considered.

There are other Euphorbiaceae species, which were less extensively studied, yet have shown some cytotoxic activity. Latex extracts from 3 plant species - *Euphorbia neriifolia* L., *Sapium laurocerasus* Desf., *Croton menthodorus* Benth - showed significant cytotoxic activity in the brine shrimp lethality test (BSLT) and DNA-methyl green interaction test (DNA-MG). However, the confidence intervals of the LC50 concentrations were relatively wide. The physicochemical analysis of another member of the Euphorbiaceae family, *Croton celtidifolius* Bail latex showed a high content of phenolic compounds. The MTT assay, showed a dose-dependent cytotoxic effect of the aqueous solution of the latex through the reduction of cell viability in MCF-7 cell lines. Isolated plasmid DNA was treated with latex supernatant, which led to DNA fragmentation. Apoptosis was visible in the majority of cells, however, membranes of the cells remained intact. The mouse model studies showed an increased median in survival time, as well as inhibited tumor growth when high latex concentrations were administered. The aforementioned results suggest the cytotoxicity of *C*. *celtidifolius* latex, which might be attributed to nuclease activity leading to direct DNA damage (Biscaro et al., 2013). It has been presumed that the cytotoxic activity may be caused by the synergistic effect of the phenolic and lignin compounds present in the latex (Biscaro et al., 2013). The hydroalcoholic extract from the latex of *Euphorbia lactea*, has been shown to be effective on the HN22 head and neck squamous cancer cell lines. It presented a significant and dose dependent cytotoxicity, decrease in the wound-closure rate and suppressed migration by 40% at most. Additional analysis showed an increase in sub-G1 population cells, indicating a blockage of the cell cycle (Wongprayoon and Charoensuksai, 2018). Administration of *Euphorbia macrolada* latex extracts on human breast adenocarcinoma cell line (MDA-MB-468) resulted in a 50% growth inhibition. The dichloromethane fraction was the most cytotoxic in this study, probably due to the presence of ingenol-type diterpenoids and triterpenoids, extracted by non-polar solvents like dichloromethane (Ravikanth et al., 2003; Corea et al., 2005; Sadeghi-Aliabadi et al., 2009).*E. heliscopia* latex contains a complex mixture of different biological substances, mainly flavonoids and diterpenoids. *Euphorbia helioscopia* ethyl acetate extract (EAE) had inhibited the growth of hepatocellular carcinoma SMMC-7721 and BEL-7402 cells. Treatment of cells with EAE decreased the amount of S-phase cells in a dose dependent manner. After treatment with EAE the amount of apoptotic cells grew and the signs of apoptosis (chromatin marginalization and condensation, cytoplasmic vacuolization, abundance of autophagic vacuoles) were also observed under the microscope. The use of the transwell chamber approach showed that EAE was able to negatively affect the invasiveness of SMMC-7721 cells. Since tumor invasion is also related to the presence of matrix metalloproteinases (MMPs), MMP-9 levels were measured and shown to be decreased in latex treated cells. (Wang et al., 2012). *In vivo* study found that ethyl acetate extract (EAE) from *E. helioscopia* L. was able to inhibit tumor growth in nude mice hepatocellular carcinoma xenografts (Cheng et al., 2015). The administration of different concentrations of EAE showed a decrease in tumor size, with the highest efficacy at 200 μg/ml latex concentration. Immunohistochemical staining also showed a reduction of CyclinD1, a cell cycle regulating protein. Electron scanning microscopy revealed changes in the morphology of tumor nuclei, including nuclear pyknosis, chromatin marginalization and condensation of chromatin as well as swelling of organelles, cytoplasmic vacuolization and presence of apoptotic bodies in EAE treated cells. Analysis of the protein expression profile suggests the arrest of cells at the G1 phase with a reduction of cells in the S phase, resulting in the inhibition of cell proliferation (Cheng et al., 2015). Apoptosi of *E. antiquorum* methanolic extracts were examined using *C. cerevisiae* cells, chick embryo fibroblasts and a brine shrimp assay (Sumathi et al., 2011). Both viability of non-cancerous cells as well as the mortality of levels of brine shrimp were decreased as the concentration of *E. antiquorum* latex increased. Chick embryo fibroblasts exposed to etoposide, a *E. antiquorum* latex component, showed nuclear apoptotic morphology (Sumathi et al., 2011) The activity of another Euphorbiaceae species, *E. neriifolia*, has been examined on Ehrlich-Lettre ascites carcinoma (EAC) and Dalton Lymphoma ascites (DLA) cancer cell lines. A cytotoxic dose-dependent effect was noted after exposure of cells to acetone extract. In an additional study, EAC and DLA cell suspensions were injected into the peritoneal cavity of mice to induce tumor formation. Further administration of *E. neriifolia* latex resulted in a significant increase in the survival period, nearly doubling the lifespan of mice treated with both low and high doses of the latex. The average lifespan of *E. neriifolia* treated mice was higher than that of the positive control mice using cyclophosphamide, which is a currently-used anticancer drug (Upadhyaya et al., 2017). Another study using the canine model has also been used in testing the activity of *Synadenium grantii Hook F.* on a preclinical prostate model. *S. grantii* in folk medicine is recommended *via* oral administration for prostate cancer patients. Two prostate cell (PC) cultures were established from intact dogs with non-metastatic PC (PC1) and with metastatic PC (PC2). All of the tested latex concentrations had an effect on both PC1 and PC2 cells in a dose-dependent manner (Brito et al., 2021). It is also crucial to note the activity of proteins present in the Euphorbiaceae latex. *Euphorbia trigona* latex is high in lectin-content, which is responsible for precipitation and agglutination in cells through binding specifically and reversibly to carbohydrates (Jawade et al., 2016). Three of those lectins have been characterized and their RNA N-glycosidase activity was tested on several human cancer cell lines, including HeLa, A549, HCT116, HL-60, human colorectal HT-29. In each sample the dose dependent decrease in activity was noticed. Flow cytometry showed phosphatidylserine (PS) externalization, which indicated the early stage of apoptosis. It is worth noting that purified isoforms were inactive in the HT-29 cell line, suggesting that certain cell lines react differently to other lectins, which might be due to differences in affinity to specific sugars present on the cell surface (Villanueva et al., 2015). This study shows that apart from small molecular weight compounds present in latex, proteins still have a significant role in potential anticancer activity of plant extracts.

There is a great viability between latex activity of latex of Euphorbiaceae species. Analysis of the latex of six *Euphorbia* species showed that despite the fact that all of the tested species contain phytosterols, triterpenoids, flavonoids and glycosides, with no proteins detected their bioactivity varied probably due to an additional compounds present in some of the plant subspecies (**Table 4**). The cytotoxic activity varied between different hexane extracts, with *E. arabica* being the most active. On the contrary, *E. gorgonis*, which lacked tannins in its latex, exhibited no antiproliferative activity, which emphasized the effects of specific latex compounds determining its biological potential. Differences between the polarity of solvents in extracts were highlighted by the non-polar extracts showing cytotoxic activity, whereas the polar extracts showed none (Tebogo Michael Mampa et al., 2020). Due to those differences, highlighted in one single study, we cannot distinguish the particular species that possess the best qualities over the other. But with the several studies (including *in vivo* studies using animal models) on Euphorbiaceae latex we can conclude that this family is especially rich in potential biopharmaceuticals and can serve us in the future.

The *Jatropha* genus is another member of the Euphorbiaceae family. Latex from the representatives of this genus have shown potential anticancer effects. *Jatropha curcas* L., a representative of this group, resides in the subtropical and tropical areas and is known for its stem-exuding latex. It has already been proven that *J. curcas* possesses antioxidant, antimicrobial, hepatoprotective, antidiabetic, and wound healing properties (Abdelgadir and Van Staden, 2013) and its latex is rich in many secondary metabolites, including phenolic compounds (**Table 4**) (Ahmed et al., 2020).

A phytochemical analysis of *J. curcas* latex revealed the presence of tannins, which are known to be used as astringents and hemostatics due to their ability to coagulate proteins in open tissues, which leads to forming a protective shield. The agar overlay technique was used to study the cytotoxic activity of *J. curcas* latex and showed the moderate cytotoxic effect, as well as necrotic coagulation. *J. curcas* latex used on L929 fibroblasts showed decolorized zones between 2-5 mm with no signs of cell lysis. Yet since cell death was visible, the authors claimed it was caused by coagulative necrosis (Siregar, 2015).

The chemosensitivity of alkaloids obtained from *J. curcas* latex was tested on human leukemic cell lines (Jurkat J6), *via* the SRB and MTT assay. The LC50 value was shown to be less than 10 μg/ml (Ag and Lele, 2013). Root extracts from *J. curcas*, as well as singular diterpenoids isolated from the latex, showed cytotoxic activity against HeLa and L5178y mouse lymphoma cells and lower activity against PC12 rat cells of neuronal origin, with concentrations below 5 μg/ml of hexane extracts being the most cytotoxic. The analyzed diterpenes, curcosone A, B and D, showed cytotoxic activity, with very low concentrations of curcusone C exhibiting high cytotoxicity (Aiyelaagbe et al., 2011). Curcusone B had already been proven to possess anticancer activities through the antimetastatic mode of action. *J. curcas* extract containing curcosone B was able to reduce tumor invasiveness in 4 human cancer cell lines (MCF-7, Hep3B, KKU-100 and KB) with Hep3B being the most sensitive to the treatment. Gelatin zymography results presi ented low undetectable levels of gelatinase (matrix-degrading enzyme) for KB, MCF-7 and Hep3B cell lines while displaying higher levels of MMP-2 activity for KKU-100 cells. Moreover; the ability to adhere to a Matrigel-coated surface was reduced. All of the above suggests a potent antimetastatic effect of curcusone B (Muangman et al., 2005). Curcin, which is a toxic protein isolated from *J. curcas*, acts as a toxalbumin, type I RIP (ribosome-inactivating protein), was shown to be involved in cell-free translation inhibition in the reticulocyte lysate, expressing cytotoxicity through inhibition of protein synthesis in gastric cancer SGC-7901, Sp2/0, HeLa and human hepatoma cell lines. The aforementioned properties imply that curcin is suitable for preparation of immunoconjugates (Lin et al., 2003). Incubation of the ribosome with curcin resulted in cleavage of rRNA, suggesting that curcin has RNA N-glycosidase activity comparable to that of trichosanthin. On the contrary, in studies of Ahmed et al. non-polar and total protein fractions did not have a cytotoxic effect, which the authors suggested was due to the presence of polar secondary metabolites. In the total methanolic fraction as well as in the samples containing curcin and saponins, the viability of the MCF-7, HCT-116 and HepG2 cell was greatly reduced, with HepG2 cells being the most sensitive (Ahmed et al., 2020). An additional *in vivo* assay on chemically induced hepatocellular cancer rat models showed that rats treated with curcin, saponins and the total methanolic extract had relatively healthy liver morphology, with the exception of a limited number of small nodules observed in a few animals. Additionally, liver functions tests revealed signs of improvement of diethylnitrosamine (DENA)-induced liver insufficient liver functions, especially for rats treated with curcin. Histological examination of the liver showed preneoplastic changes with disarranged and enlarged hepatocytes in methanolic extract-treated rats (Ahmed et al., 2020).

Studies using mouse bone marrow erythrocytes supported previous findings, which indicated that *J.curcas* latex can induce cytotoxic and mutagenic potential in mammalian cells (Bailão et al., 2019). Surprisingly, co-administration of the latex along with doxorubicin, a common chemotherapy drug, reduced the normally-observed induced side effects. This is presumably due to *J. curcas* latex showing protective activity towards cells through scavenging free radicals. It is worth noting that since herb-drug interactions are not well inspected, such treatment can result in reduction of drug activity (Bailão et al., 2019). To gain more data on the safety of *J. curcas* latex use, cytotoxicity of 37-10.000 μg/ml latex was evaluated by the MTT assay. Results showed that from the 2 cell types studied - Fib L929 and human gingival fibroblasts - Fib L929 was more resistant to the latex (Siregar and Akbar, 2000). To further establish the genotoxic and cytotoxic potential of *J. gossypiifolia* latex the *A. cepa* test was used. 1.25, 2.5 and 5 ml/L of latex led to chromosome adherences, chromosome bridges, C-metaphases, decrease in root mean growth and lowered the mitotic index in a dose dependent manner. Moreover, in all analyzed treatments polyploid, binucleated cells, nuclear buds and nobulated nuclei have been found along with non-significant alterations, such as chromosome losses or mulipolarities. These findings suggest that the use of this latex can be harmful to human health, although the concentration of the used latex was high compared to other studies on the topic (de Almeida et al., 2015). The *A. cepa* model confirmed the finding for previously mentioned *J. curcas*, whose crude latex and its 50% dilutions had affected the germination process of *A. cepa*, inhibited the mitotic index (MI) of root cells and induced cytotoxicity. The chromosomal aberrations (chromosome bridging, lagging chromosomes and chromosome stickiness) were observed with low incidence with low latex concentrations used (1%, 0.5%) and increased drastically with a 0.01% concentration (Ciappina et al., 2017). As the finding suggests, *J. curcas* latex, with the focus on curcosone and curcin, is a valuable source of potentially anticancer molecules although the molecular mode of action is not yet fully known and further genotoxicity tests must be conducted.

### Family: Moraceae

Few latex-bearing plants have been studied from the Moraceae family, with the majority of studies focusing on the *Ficus* genus. This genus is well known for its medical properties and has been used as a chemoprotective agent, due to its anti-acne and anticancer properties. Its barks have been used to treat menorrhagia, leucorrhea, diarrhea and urogenital disorders while the roots have been used in vitiligo and ringworms (Nadkarni and Nadkarni, 1994; Tulasi et al., 2018). *Ficus* latex is particularly rich in phytosterols (**Table 5**), as well as in amino acids and fatty acids. It is most known for its fig fruits, which are rich in polyphenols, anthocyanins and flavonoids due to which they own their anti-inflammatory and antiparalytic properties (Nadkarni and Nadkarni, 1994; Solomon et al., 2006).

**Table 5:** Biological compounds with potential anticancer activity present in the latex from Moraceae family representatives.

Plant species	Compounds present in the latex		Reference
	Low molecular weight molecules	Proteins	
*Antiaris toxicaria*	Cardenolide glycosides		(Dai et al., 2009)
*Ficus carrica*	6-O-acyl-β-D-glucosyl-B-sitosterols (palmitoyl, linoleoyl, oleyl and stearyl) Amino acids Fatty acids Phytosterols (B-sitosterol, α and β-amyrin, lupeol, betulol and lanosterol)	Cysteine proteinases (ficin)	(Rubnov et al., 2001; Solomon et al., 2006; Hashemi et al., 2011)
*Ficus religiosa*	Flavonols (quercetin and myricetin) Phytosterols (stigmasterol and β-sitosterol)		(Saida et al., 2021)
*Ficus pseudopalma*	Flavonoids (quercetin) Terpenoids (lupeol)		(De Las Llagas et al., 2014)

*Ficus religiosa* is a representative of the *Ficus* genus with well documented cytotoxicity against cancer cell lines, including human neuroblastoma (IMR 32), human colorectal cancer (HCT 116) and human breast adenocarcinoma (MDA MB 231), as well as on human peripheral blood mononuclear cells (PBMNCs). The cytotoxic activity is mainly attributed to the high flavonoid content in its latex. Prominent morphological changes like loss of shape, disruption of the nuclear membrane and detachment of cells from the culture plate bottom were noticed, along with inhibition of cell growth and cell death after 24 h. Additional studies of apoptotic genes showed changes in their expression (**Table 3**). Flow cytometry also showed cell arrest in the C1 and C2/M phase cancer cell lines treated with latex extracts (Saida et al., 2021). *F. religiosa*, along with *F. benghalensis*, was also effective against MCF-7, leading up to 90% growth inhibition with 200 μg/ml concentration used (Tulasi et al., 2018). Crude ethanolic extract, as well as ethyl acetate and chloroform fractions, from *Ficus pseudopalma*, an endemic Philippine tree, whose latex is rich in compounds like lupeol and quercetin showed strong concentration-dependent inhibitory activity against PRST2 cells and higher cytotoxicity towards prostate cancer and HepG2 cells (De Las Llagas et al., 2014). *F. carica* whole latex also showed apoptosis-inducing and antiproliferative activity towards HCT-116 and HT-29. The antiproliferative activity was more prominent in ethyl acetate extract treated cells. HT-29 was less responsive to treatments, this is presumed to be caused by its being a more aggressive cell line and differences in invasiveness, hence different responsiveness to treatment can occur (Soltana et al., 2019). The cytotoxic effect of *F. carica* latex has also been confirmed on stomach cancer cell lines. 72 hour treatment of cells with different latex concentrations inhibited the growth of the cancer cells, but bearing no toxic effect on the control PBMNCs. Such activity may be due the presence of ficin - cysteine proteinase known to lead to cancer cells apoptosis as well as the presence of polyphenolic compounds. This suggests that both polar and non-polar latex fractions from various Ficus species can possess potential anticancer activities.

Apart from using crude latex or its whole fractions it is again crucial to detect singular compounds responsible for biological effect. The cytotoxic activity of several compounds from *Ficus carica*, including palmitoyl, linoleoyl, oleyl and stearyl as well as derivatives of β-sitosterol-β-D-glucoside, was tested on 6 cells lines including two lymphoma Burkitt B cell lines (Raji and DG-75), T-cell leukemia cell lines (Jurkat, HD-MAR), prostate cancer cell lines (DU-147) and MCF-7 and showed cytotoxic effects in a time and dose-dependent manner. Synthetically prepared derivatives expressed similar activity to that of natural compounds (Rubnov et al., 2001; Hashemi et al., 2011). *F. carica* had also been acting cytotoxic against HeLa cell line, reducing viability of the cells at concentrations as low as 2 μg/ml. All used extracts (ethanol, ethyl acetate, dichloromethane) as well as crude latex had similar moderate activity with IC50 values between 10-20 μg/ml which corresponds with the results obtained from *F. religiosa* (Khodarahmi et al., 2011). Administration of *F. carica* latex on human cervical cancer cell lines HeLa (HPV type 18) and CaSki (HPV type 16) had shown the presence of active lipophilic anti-HPV compound being a ferulic/caffeic acid/chlorogenic plant sterol derivative. The affected cells also showed signs of exit contact inhibition, which is a feature of cancer cells along with misplacement of Ki67 protein, suggesting that fig latex targets the expression of Ki67 playing a role in preventing cell proliferation. Fig latex also was able to downregulate the expression of HPV oncoproteins, which can promote metastasis and cell invasion (**Table 3**) (Ghanbari et al., 2019). Fatma Mousa AlGhalban et al. compared the activity of latex of *F. carica* and less studied *Ficus* specie *F. salicifolia*, native to the United Arab Emirates and used traditionally to treat cough, chest inflammation, scorpion stings and bruises (Gushash, 2006). It was found to act antiproliferative as well as antimetastatic with just 0.1% of concentration of latex used resulting in stress apoptosis in MDA-MB-231-triple-negative-breast cancer cell lines. Moreover, the latex led to reduced cell proliferation as well as abnormal morphology including shrunk in spindle shape, cell blebbing and cytoplasmic vacuolation. Nucleus blebbing and crescent shape suggested different stages of apoptosis occurring. The scratch wound healing assay has shown the reduction in cell migration in both species even at 0.01% concentration used, while phosphokinase array kit has shown changes in expression of genes involved in cell proliferation and cell attachment in *F. carica* leaf latex. Interestingly, *F. salicifolia* leaf latex increased expression of all genes (**Table 3**) (AlGhalban et al., 2021).

Not only latex or its extracts can act cytotoxic. Jing Wang et al. tested the activity of fig fruit latex (FFL) on SMMC-7721, human glioblastoma (U251) and normal liver cells (L02) and found it to exhibit potent cytotoxicity against cancer cells whilst not affecting normal cells. Additionally, inhibitory dose-dependent colony-forming effects were observed as well as significant increase in the number of apoptotic cancer cells with increase in number of cells in G0/G1 phase and decrease in S and G2/M phase cells. In the case of healthy liver cells the effect was reversed. Hence the anticancer activity of fig fruit latex is thought to be based on inhibition of DNA synthesis as well as proliferation and apoptosis induction (Wang et al., 2008).

Apart from Ficus genus there are other representatives of the Moraceae family including *Antiaris toxicaria* which has recently gained interest. The latex of this plant is highly toxic, this trait is thought to be founded on the presence of cardenolide glycosides within (Dai et al., 2009). A compound isolated from *A. toxicaria* showed inhibiting growth activity in several cancer cell lines, with IC50 values ranging between 0.004-0.037 μg/ml (Dai et al., 2009). However; due to the great number of Ficus genus studies we assume that currently this genus has gained the most attention and will be studied more in the future.

### Family: Papaveraceae

A representative of the latex-bearing plant family Papaveraceae, which possesses anticancer properties, is the widely distributed plant *Chelidonium majus* L. This plant has been used in folk medicine for centuries and has gained attention due to its probable anticancer effects as well as antifungal, antibacterial and immunomodulatory properties (Gracz-Bernaciak et al., 2021; Nawrot et al., 2021). The latex of this plant is rich in alkaloids (Zielińska et al., 2018), to which the antitumor properties are mostly attributed to. The pharmacologically important compounds (reviewed in (Maji et al., 2015) include chelidonine, sanguinarine, chelerythrine and berberine. The aforementioned alkaloids can act through the induction of apoptosis, as well as upon DNA through intercalation, which results in the disruption of replication and cell division (Jyoti, 2013; Zielińska et al., 2018). This in turn causes limitation and prevention of cancer growth.

**Figure 2:**
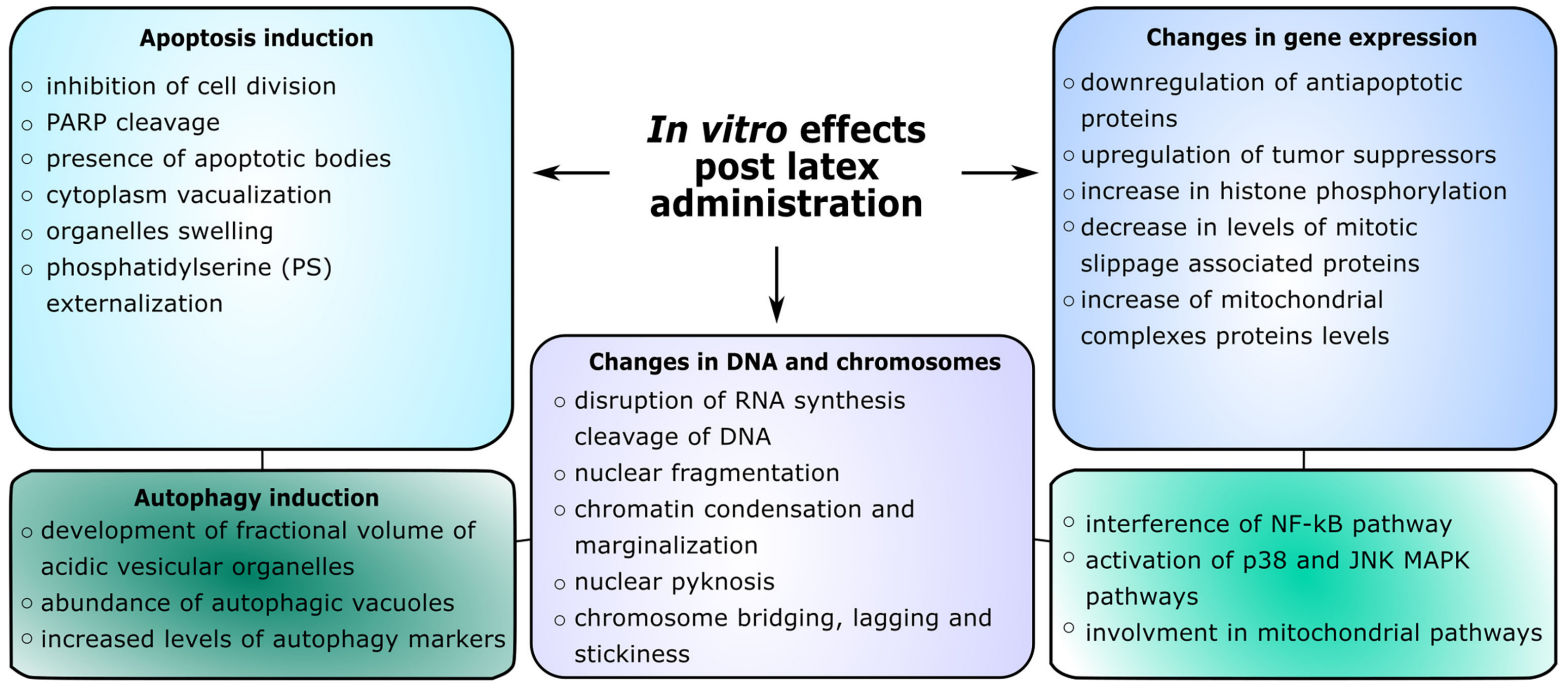
Overview of *in vitro* effects on cells after administration of plant latex extracts or its compounds.

Several alkaloids, which have been isolated from *C. majus* latex, were studied in their effects towards various cancerous cell lines, including leukemias, melanomas, colon, breast, pancreatic, lung, gastric, Hela and prostate cancer cell lines (2008; 2017; Panzer et al., 2001; Kaminskyy et al., 2006; Kemény-Beke et al., 2006; Lee et al., 2007; Philchenkov et al., 2008; Vrba et al., 2008; Noureini and Wink, 2009; Hammerová et al., 2011; Paul et al., 2013a; Zhang et al., 2014; Capistrano I et al., 2015; Kim et al., 2015; Deljanin et al., 2016; Havelek et al., 2016; Qu et al., 2016; Noureini et al., 2017; Zielińska et al., 2018). The outcome of the action upon various cell lines was correlated with the cell type and varied - some alkaloids showed strong observed responses, whereas others showed weaker. Several studies showed cytotoxic effects towards cancer cells with the use of a single alkaloid towards the cell, however, some studies included a mixture of alkaloids (Kulp et al., 2011; Kulp and Bragina, 2013) or nano-encapsulated alkaloids (Paul et al., 2013a). What is more, due to the variations in the alkaloid composition in *C. majus* latex, the mechanisms of the cytotoxic effect are very diverse, especially when taking into consideration different cell death signaling pathways (Philchenkov et al., 2008) (**Figure 2**). A study using capillary electrophoresis was carried out to measure the effects of a mixture of *C. majus* alkaloids on murine fibroblast cells NIH/3T3, B16F10 and MCF-7. The cytotoxic activity was inversely proportional to the ability to penetrate cells, the greatest ability was observed for alkaloids in the NIH/3T3 cell line, however, the greatest cytotoxic effect *via* apoptosis was observed in the B16F10 line (Kulp and Bragina, 2013). Therefore, the combination of five alkaloids showed an apoptotic effect on cancer cells. Additionally, the effect of *C. majus* plant extract through indirect action on cancer cells was studied. Post exposure to the *C. majus* extract the PBMNCs showed greater cytotoxicity towards Helacells in comparison to untreated PBMNCs. The extract itself was not toxic to the PBMNCs. These findings suggest the immunomodulatory effect of the *C. majus* extract (Popovic et al., 2022). In primary endometrial cancer cells a low to no cytotoxic effect was observed when treated with 4 main alkaloids from *C. majus* (Capistrano I et al., 2015)

Chelidonine is one of the most studied alkaloids present in *C. majus*. (Panzer et al., 2001; Kemény-Beke et al., 2006; Kaminskyy et al., 2008; Philchenkov et al., 2008; Hammerová et al., 2011; Qu et al., 2016)Results from both MTT assays and the assessment of phosphorylation levels of histone H3 at Ser^10^ (**Table 3**) show that chelidonine inhibits proliferation and induces arrest of cells in the M phase, respectively (Qu et al., 2016), which in turn cause an increase in the number of apoptotic cells over time. Moreover, the particles did not show any toxicity profiles in mice. The effect of chelidonine was also tested on pancreatic cancer cell lines (BcPC-3 and MIA PaCa-2) and showed an apoptotic outcome (El-Readi et al., 2013; Paul et al., 2013b; Maji et al., 2015). In both aforementioned cell lines p53 was found to be upregulated and a Western blot analysis after a 24 hour incubation showed the increase of arrest-related proteins (**Table 3**) (Jang et al., 2021), and was additionally re-confirmed in a loss of function study using siRNA against p53 and GADD45a (Jang et al., 2021). Other alkaloids, including sanguinarine, dihydrochelerythrine, protopine, berberine and nitidine, show anticancer properties by induction of apoptosis by activation of caspase 3 and 9, dissipation of the mitochondrial membrane potential and upregulation of proapoptotic proteins (detailed description summarized in (Maji et al., 2015). The aforementioned alkaloids were also tested for their apoptogenic and DNA damaging effects in MT-4 cells (Philchenkov et al., 2008). In comparison to chelidonine, which showed low DNA-binding capacity, sanguinarine showed high DNA intercalation ability (Philchenkov et al., 2008). Despite these differences, both alkaloids showed apoptogenic activity in MT-4 cells in a comet assay, through apoptotic bodies and chromatin fragmentation in the case of sanguinarine and mitotic impairment in the case of chelidonine (Philchenkov et al., 2008). The aforementioned studies highlight the importance of apoptosis induction as a mode of cancer treatment and provide a possible candidate to carry this out, since the apoptotic effect of chelidonine towards cancer cells has been confirmed. Contrastingly to other findings, the extract from rhizomes and roots of *C. majus* showed no activity towards L-1210 leukemia and Walker 256 intramuscular carcinosarcoma cells. This can indicate that the latex itself can provide ample sources of anticancer substances.

Chelidonine and its dimethoxy analogue were studied for their cytotoxicity against human leukemic (including Jurkat and MOLT-4 cells) and lung cancer cell lines (Havelek et al., 2016). The investigation of the anticancer activity against human blood cancer cell lines was performed *via* trypan blue exclusion. Homochelidonine, as well as chelidonine, were able to decrease the proliferation of Jurkat and MOLT-4 cells, with MOLT-4 cells being more resistant to treatment. The mitochondrial membrane potential measurements showed that in both Jurkat and MOLT-4 cell lines, administration of chelidonine and homochelidonine resulted in the decrease of cells with intact mitochondria. Additionally, both chelidonine and homochelidonine showed inhibitory effects towards the proliferation and adhesion degree of A549 cells, which was determined by real time xCELLigence analysis (Havelek et al., 2016).

*C. majus* latex fractions were shown to decrease the viability at least by 50% of HeLa (HPV-positive) and C33A (HPV-negative) cell lines. The results suggested that fractions containing the major latex protein (CmMLP1) along with alkaloids showed the highest cytotoxic activity against HPV-positive cells (Nawrot et al., 2021).

Other components of the latex, which possess anticancer properties, include proteins such as a protein-bound polysaccharide (CM-AIa) and lectin, which showed immunomodulatory, cytotoxic and growth inhibitory activity of cancer cells *in vitro* (Fik et al., 2001; Song et al., 2002; Maji et al., 2015; Zielińska et al., 2018). Additionally, studies of two particularly abundant latex nucleases (CMN1 and CMN2) isolated from the milky sap of *C. majus* were tested on HeLa and Chinese hamster ovarian cells. After a 48h incubation of protein fractions with the CHO cells no effect was noted, however; incubation of nucleases with HeLa cells resulted in the presence of apoptotic lesions in a dose-dependent manner. What is worth mentioning is that the purified form of the nucleases varied in activity between the seasons during which the latex from the plant was collected - the highest activity was visible in the May fractions and the lowest in October (Nawrot et al., 2008). This variance in activity may be attributed to different post-translational modifications of the enzymes in different months of the year, as well as changes in the presence of cofactors in the latex at different vegetation period time points (Nawrot et al., 2008).

Data regarding the clinical use of *C. majus* latex compounds is scarce. Discontinuation of *C. majus* administration showed a favorable clinical outcome. In a case study, during which the B-cell chronic lymphatic leukemia patient was treated with the RNA polymerase inhibitor amanitin, isolated from *Amanita phalloides*, a decrease of thrombocyte levels was observed, along with a necessary increase in the dosage of the applied treatment. Due to its cytotoxic potential, the extract from *C. majus* was additionally given to the patient. The results showed that although *C. majus* had no strong effect on tumor growth of cells, the level of thrombocytes was increased, which indicated a positive effect on the bone marrow (Riede, 2016). Moreover; Ukrain, which was a compound broadly tested and had promising results, however; a systematic review conducted by E Ernst and K Schmidt had revealed significant malfunctions in the clinical studies on Ukrain and suggested more in-depth studies before any strong conclusion could be stated (Ernst and Schmidt, 2015). Nevertheless, the latex of *C.majus* has potentially a fair number of compounds which might be proven useful and.

## Latex-bearing plants and nanotechnology

### Nanomaterials as metabolites delivery vehicles against cancer

Nanotechnology has gained great interest in the biomedicine area. Nowadays nanostructures, including nanoparticles (NPs) and various nanomaterials, are the most promising tools for the development of new strategies in medicine. Due to their unique physicochemical characteristics (size, morphology, chemical properties) nanostructures have medical applications in several/numerous important fields. Nanomaterials can be applied as nano carriers for drug delivery (Mitchell et al., 2021) and as contrast agents (Caspani et al., 2020). Additionally, nanostructures are suitable candidates for bioimaging (Gil et al., 2021), theranostics (Madamsetty et al., 2019) and tissue engineering (Hajialyani et al., 2018) as well as cancer diagnostics and therapy (Singh et al., 2022).

The increasing need for effective delivery of antitumor drugs has triggered the development of nanotechnology-based systems. The special feature of nanoparticles is an extremely high surface area to volume ratio, which allows them to successfully bind small-weight molecules (Tyavambiza et al., 2021). Thus, in recent years, the combination of plant-derived compounds with nanomaterials has gained enormous popularity. Numerous types of bioactive low-molecular weight metabolites present in plant latex such as alkaloids, flavonoids, have been effectively associated with nanomaterials (Mehan et al., 2022). Several studies revealed that nanoencapsulation of pharmacologically active plant phytochemicals can increase the therapeutic efficiency in cancer treatment (de Alcantara Lemos et al., 2021). Nano-formulatons are notably important for effective delivery of poorly soluble and low-bioavailable pharmaceuticals. Biofunctionalization of nanomaterials with natural plant compounds improves solubility, increases bioavailability of natural compounds and reduces their potential cytotoxicity (Yang et al., 2020; Vanti, 2021). Moreover, encapsulation of bioactive molecules allows controlled release of drugs, thus improving the therapeutic effect. Hesami et al. showed anticancer activity of chitosan NPs loaded with essential oils from *C. majus*. This group reported a strong cytotoxicity of loaded NPs against MCF-7 cells. Moreover, treatment of MCF-7 cells with chitosan NPs induced apoptosis related pathways (Hesami et al., 2022). Another study indicated that polysaccharide-based NPs can be used for loading natural plant compounds. Berberine, one of the alkaloids that exists in milky sap of *C. majus*, was loaded to biocompatible chitosan-alginate based NPs. Berberine possesses strong biological activities (including anticancer effects) and low toxicity, but is a poorly absorbed drug (bioavailability in oral administration in rats is <5%). This limitation can be overcome by using nanosystems. Kohli et al. reported that loading berberine into polisachaccharine-based NPs improved its intestinal absorption. Furthermore, formulated chitosan-alginate NPs loaded with berberine constitute a fully biocompatible and biodegradable system (Kohli et al., 2021).

Moreover, active natural constituents can be easily incorporated into polymeric nanomaterials (Huesca-Urióstegui et al., 2022). Recent studies revealed that pharmacologically active small-weight molecules from *C. majus* can be effectively combined with natural collagen. Generated antibacterial plant-based collagen composites possess antimicrobial properties and can be potentially used in treating skin wounds to facilitate the wound healing process (Warowicka et al., 2022). Furthermore, Mouro et al. presented that crude *C. majus* extract was effectively incorporated into polymeric nanofibers. The produced Polycaprolactone (PCL)/Polyvinyl Alcohol (PVA)_Pectin (PEC) nanofibers matrices, which contained *C. majus* constituents, prevented bacterial wound infections and improved the healing process (Mouro et al., 2021; Tyavambiza et al., 2021). The sap from the stem and leaves of *Ficus asperifolia*, and leaf gel from *Aloe vera* and *Aloe ferox* also possess wound healing activities (Tyavambiza et al., 2021). *A. vera* extract has been incorporated into chitosan/polyethylene oxide (PEO) nanofibers by electrospun. Other than antioxidant properties, these composites have been shown to have anticarcinogenic properties (Hajialyani et al., 2018; Pathalamuthu et al., 2019). Moreover, the extract from *Acalypha indica* L. (Euphorbiaceae), which is rich in alkaloids, flavonoids, and saponins, can be used in nano-formulations. This herb possesses anti-inflammatory and antioxidant properties, thus can be considered also in anticancer studies (Qadir et al., 2021).

### Nanoparticles synthesis with the use of latex-bearing plants

The next hot topic, which is booming in nanotechnology and is extensively studied, is green-synthesis of NPs. Green-formed nanoparticles exhibit beneficial biological properties, such as anticancer, anti-inflammatory and antimicrobial activities (Jain et al., 2021). It is worth noting that anticancer activity of NPs depends on their size and shape. Studies showed that smaller nanoparticles can easily penetrate tumor tissue. Interestingly, ultrasmall-sized gold NPs (<10 nm) can cross the nuclear membrane of breast cancer cells (Huo et al., 2014). Previous studies have suggested that plant extract-assisted synthesis of NPs can be successfully applied to generate NPs in a controlled small and ultrasmall size range 2nm-10nm (Yang et al., 2019; Jeevanandam et al., 2020). Moreover, several studies have shown that plant-based generated NPs possess excellent optical, magnetic or mechanical properties (Dinkar et al., 2021).

**Figure 3:**
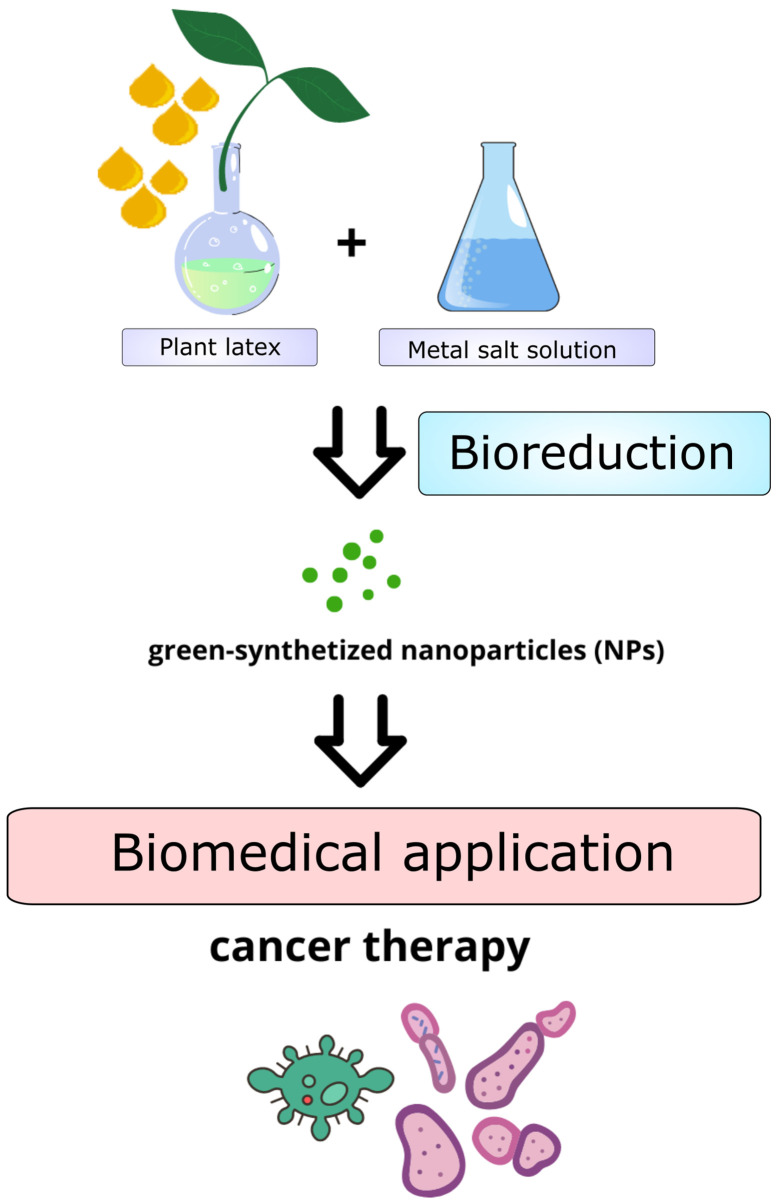
Schematic overview of the preparation of green-synthetized nanoparticles (NPs) using plant latex.

Medicinal latex-rich plants have been used for biological synthesis of different types of a wide range of nanomaterials including metallic NPs, polymer NPs, carbon NPs and nanocomposites. Numerous studies have addressed biogenic formation of metallic NPs, such as silver (AgNPs), gold (AuNPs) or metal oxide NPs, including zinc oxide, copper oxide and iron oxide. It is well known that plant latex is composed of polar and non-polar molecules. Inorganic biological components, such as alkaloids and flavonoids, are useful in the synthesis of NPs by reducing metal ions (Shafey and El Shafey, 2020; Adeyemi et al., 2022). In plant-mediated synthesis plant natural compounds are applied as bioreducing, capping and stabling agents (Ranabhatt and Kapor, 2017; Chopra et al., 2022). In particular, plants with the capability to accumulate heavy metals in their distinct parts are used for green-synthesis of NPs (**Figure 3**). In comparison to chemical synthesis, formation of nanoparticles by eco-friendly, biological models has numerous advantages over traditional/classical/currently used methods. Green-synthesized NPs are generated without the involvement of toxic, harmful chemicals, are low-cost procedures, use biologically safe solvents and safe, easily available plant material. Moreover, phytosensitized NPs are stable, biocompatible, and potentially less toxic. Such characteristics make green-synthesized NPs attractive materials for biomedical and commercial applications.

Recently, extensive attention has been directed towards plants bearing latex for the formation of NPs (*Euphorbium* sp., *C. majus*, *Aloe* sp.). Natural constituents from latex of Euphorbiacea family plants were utilized as an bioreducing agent for the fabrication of AuNPs (Lunardi et al., 2018). Thus, metal ions were reduced to nanoparticles. Physicochemical characteristics reveal that AuNPs synthesized using *E. tirucalli* latex are spherical in shape and polidyspersed. Thus, dried latex from *E. tirucalli* has attracted great attention owing to their properties and the potential application in nanotechnological drug delivery systems. Valodar et al. reported that the stem latex from *Euphorbia nivula* was successfully used to rapid synthesis of silver and copper NPs (Valodkar et al., 2011). Kameswari et al. showed anti-inflammatory and antioxidant activities of selenium NPs synthetized using *A.indica* L. latex plant (Euphorbiaceae). Another study demonstrated that biogenic selenium nanoparticles synthesized by *Aloe vera* extract exhibited antioxidant activity, including them as potential NPs in various disease therapies (Vyas and Rana, 2017). Moreover, *Aloe vera* extract was used as a reducing agent for synthesis of gold and silver NPs (Chandran et al., 2006). Interestingly, the plant extract-assisted synthesis allowed control of the morphological parameters of metallic NPs (e.g. shape and size). Additionally, *Aloe vera* extract was used to produce tellurium nanoparticles (TeNPs) with potential anticancer activities (e.g. towards melanoma cells) (Medina-Cruz et al., 2021). Extract derived from *C. majus* was used to produce zinc oxide nanoparticles (ZnO NPs). Apart from the confirmed antimicrobial activity of biologically synthesized ZnO NPs, results presented by this group indicate that the ZnO NPs possess antiproliferative effects on the A549 cancer cell line (Dobrucka et al., 2017). The next example of a plant, the extracts of which were used for biosynthesis of nanoparticles, is *F. carica* (Moraceae), which contains a white milky latex. The extract from the leaves of this plant was utilized for production of AgNPs (Valodkar et al., 2011; Jacob et al., 2017) and AuNPs (Patil, 2020). Furthermore, metal oxide nanoparticles (e.g. copper oxide nanoparticles; CuONPs) fabricated by the extract from *F. religiosa*, inhibit growth of pathogenic bacteria strains in wounds such as *Staphylococcus aureus*, *Escherichia coli*. Sankar et al. also reported that *F. religiosa* extract-synthesized CuONPs increased the growth of fibroblasts and facilitated wound healing in Wistar rats (Sankar et al., 2015).

Medicinal plants, including *Calotropis gigantea*, commonly called swallow wort or milkweed, and *Euphorbia antiquorum*, were used for the synthesis of nanocarriers for natural constituent (curcumin) delivery (Samrot et al., 2019). Hence, the natural ingredients from plant latex can be encapsulated into nanosized biogenic structures.

To sum up, nanocarriers with natural herbal drugs can enhance the bioavailability of plant ingredients, improve the therapeutic effect and offer control drug release. Due to the pharmacological properties, natural products can be incorporated into polymeric materials to create bioactive and biocompatible matrices, and wound dressings. Furthermore, latex-bearing plants are a rich source of bioactive molecules which can be utilized for the biosynthesis of NPs. For these reasons, these latex bearing plant-based nanosystems can be used in various biomedical applications such as anticancer therapy, bioimaging and tissue engineering.

## Biotechnological approaches in the enhanced production of secondary metabolites

Latex-bearing plants due to their unique properties have been used for ages in the treatment of various diseases. Ancient preparations were based on wildly grown plants, however nowadays obtaining various phytochemicals is enhanced by modern biotechnology cultivation methods including *in vitro* cultures.

Such methods ease the production when no chemical synthesis is possible or large scale production would be problematic. Additionally, it allows gaining access to phytochemicals without the influence of environmental or geographical factors and with a significantly lower production time and costs (Sharma et al., 2020; Ahmad et al., 2013).

*In vitro* plant tissue culture techniques include hairy root, callus or suspension cultures as well as the use of micropropagation and bio-transformation. New approaches, including immobilization techniques, are also in demand (Wilson and Roberts, 2012). For the last 30-40 years isolated roots of medicinal plants, including latex bearing plants, have been used to produce various phytochemicals, including sanguinarine, berberine or paclitaxel (Nielsen et al., 2019). Crucial factors which need to be taken into consideration when establishing an *in vitro* culture include: medium optimization (e.g. the Murashige and Skoog (MS) medium is used in alkaloid production), the choice of a highly producing strain and the adjustment of the culture conditions, including temperature (Abranches et al., 2005). The conditions might vary depending on the culture type. For example, root cultures of some higher plants are able to generate much bigger amounts of secondary metabolites. With a well established culture, it is possible to upscale the production using bioreactor systems (e.g. feed batch, batch and continuous cultures). To further stimulate the production of secondary metabolites, elicitors (both abiotic and biotic) can be added. The treatment with an abiotic elicitor, UV-B, led to increased formation of polyphenol-rich sprouts, as well as increased flavonoid content (Hassan et al., 2008; Sharma et al., 2020). Another approach in *in vitro* cultures is based on biotransformation. It enables the synthesis of phytochemicals through the addition of a precursor into the medium and transformation of the substrate through metabolic pathways into the final product. For example, cardiac glycoside digoxin is a product of transformation of β-methyldigitoxin to β-methyldigoxin (Singh and Sharma, 2020).

With the expansion of knowledge on the metabolic pathways of various phytochemicals, more genetic manipulation techniques have been introduced to enhance the production of plant secondary metabolites. Genetic techniques may include the use of microorganisms, such as *Agrobacterium rhizogenes* or *Agrobacterium tumefaciens* which facilitate mutagenesis and allow for root transformation (Smetanska, 2008). Recently, the introduction of the CRISPR/Cas9 system (clustered regularly interspaced short palindromic repeats) allows for the simultaneous introduction of multiple edits in the genome. Novel engineering techniques allow for genome editing through deletion, insertion, modification of gene transcriptions, mutation of nucleotides and the use of RNA interference in order to cause regulatory changes in the target genome (Barrangou et al., 2007; Xu et al., 2020). However, lack of transformation protocols for non-model organisms and screening for positive clones can be a potential limiting factor in these genetic manipulation techniques (Bhagwat et al., 2022).

The aforementioned biotechnological techniques ease the production of pharmaceutically important phytochemicals at the industrial level, as well as expand the knowledge about plant metabolism at the molecular level.

## Discussion

Based on the presented data we can conclude that plant latex might be a valuable source of several active ingredients, useful in potential anticancer therapies. Their biomedical properties include antiapoptotic, antiproliferative, antiangiogenic activities as well as an effect on gene expression patterns and the transcriptome. Additionally, *in vivo* studies confirmed the ability of plant latex-based extracts or their compounds to reduce the tumor volume and its mass whilst exceeding the lifespan of mice. We assume that this makes them a promising tool in anticancer treatment, based on the aforementioned traits.

Apart from the benefits, it’s crucial to note the potential side effects, which make latex-bearing compounds in cancer treatment a challenge, mainly caused by the low specificity of the plant-based drugs, resulting in an insufficient level to target neoplastic sites (Soares de Oliveira et al., 2007). Additionally, plant sourced pharmaceuticals suffer from poor solubility, instability depending on pH levels, low absorption and fast excretion, which leads to low bioavailability and a scarce clinical effect. In order to enhance the activity of such compounds multiple delivery platforms have been designed. Polymeric nanoparticles, liposomes and micelles as well as many others can help to guide therapeutics to specific tumor sites (Dehelean et al., 2021). Examples of already used drug delivery platforms include Abraxane, a protein albumin, which is attached to docetaxel, a chemotherapeutic derived from *Taxus brevifolia* or Doxil which is a liposome-based approach with doxorubicin wrapped inside (Markman, 2011; Zhao et al., 2015). Current advancements in nanoscience have resulted in new strategies in drug formulation. In this review we described the wide use of nanosystems for successful encapsulation of bioactive plant compounds. Nowadays, the integration of natural products with nanomaterials has an extraordinary impact on targeted drug therapies as well as tissue and material engineering. Moreover, utilizing numerous plant constituents, which can act as reducing and capping agents, contribute to the development of green nanotechnology.

Other than molecular challenges and difficulties in drug delivery, there is still a strong variety in latex content not only between singular samples but also amongst entire populations, depending on their geographical location. In order to assure that the drugs based on natural origin are working efficiently, proper standardization of latex content must be conducted whilst the effective dose and concentration of singular compounds must be established. The analysis of the chemical character of secondary metabolites in natural settings can ease the discovery and production of novel synthetic or hemi-synthetic compounds with enhanced favorable effects regarding their anticancer activity. Latex-bearing plants and their valuable latex have served as a promising source of biopharmaceuticals for centuries. As we continue to discover the molecular mechanisms behind the activity of various latex-derived compounds, we propose that such drugs of latex origin can in the near future serve us to treat several diseases, including cancer. In order to assure the effectiveness and safety of latex based drugs, more studies, with the focus on clinical trials must be conducted.

## Author contributions

OM, SB, and RN contributed to the conception and visualization of the article. OM and SB organized the database. OM, SB, RN, and AW were involved in preparation and editing. OM, SB, RN, and AW wrote sections of the manuscript. RN supervised the writing process. SB, OM, and AW prepared the visual figures. All authors contributed to the article and approved the submitted version.

## Funding

This research was funded by the National Science Centre, Poland (grant number 2019/35/B/NZ9/03851).

## Conflict of interest

The authors declare that the research was conducted in the absence of any commercial or financial relationships that could be construed as a potential conflict of interest.

## Publisher’s note

All claims expressed in this article are solely those of the authors and do not necessarily represent those of their affiliated organizations, or those of the publisher, the editors and the reviewers. Any product that may be evaluated in this article, or claim that may be made by its manufacturer, is not guaranteed or endorsed by the publisher.
